# Using droplet digital PCR to analyze *MYCN* and *ALK* copy number in plasma from patients with neuroblastoma

**DOI:** 10.18632/oncotarget.19076

**Published:** 2017-07-07

**Authors:** Marco Lodrini, Annika Sprüssel, Kathy Astrahantseff, Daniela Tiburtius, Robert Konschak, Holger N. Lode, Matthias Fischer, Ulrich Keilholz, Angelika Eggert, Hedwig E. Deubzer

**Affiliations:** ^1^ Department of Pediatric Hematology, Oncology and Stem Cell Transplantation, Charité–Universitätsmedizin Berlin, Berlin, Germany; ^2^ Translational Radiation Oncology Research Laboratory, Department of Radiooncology and Radiotherapy, Charité–Universitätsmedizin Berlin, Berlin, Germany; ^3^ German Cancer Consortium (DKTK), Partner Site Berlin, Berlin, Germany; ^4^ Department of Pediatric Hematology and Oncology, University Medicine Greifswald, Ferdinand-Sauerbruch-Straße, Germany; ^5^ Department of Pediatric Hematology and Oncology, University Hospital Cologne, Cologne, Germany; ^6^ Center for Molecular Medicine Cologne, University of Cologne, Cologne, Germany; ^7^ Max Planck Institute for Metabolism Research, Cologne, Germany; ^8^ Charité Comprehensive Cancer Center, Charité – Universitätsmedizin Berlin, Berlin, Germany; ^9^ Berlin Institute of Health (BIH), Berlin, Germany; ^10^ Junior Neuroblastoma Research Group, Experimental and Clinical Research Center (ECRC), Berlin, Germany

**Keywords:** intratumor heterogeneity, liquid biopsy, non-invasive biomarker, pediatric cancer, tumor dynamics

## Abstract

The invasive nature of surgical biopsies deters sequential application, and single biopsies often fail to reflect tumor dynamics, intratumor heterogeneity and drug sensitivities likely to change during tumor evolution and treatment. Implementing molecular characterization of cell-free neuroblastoma-derived DNA isolated from blood plasma could improve disease assessment for treatment selection and monitoring of patients with high-risk neuroblastoma. We established droplet digital PCR (ddPCR) protocols for *MYCN* and *ALK* copy number status in plasma from neuroblastoma patients. Our ddPCR protocol accurately discriminated between *MYCN* and *ALK* amplification, gain and normal diploid status in a large panel of neuroblastoma cell lines, and discrepancies with reported *MYCN* and *ALK* status were detected, including a high-level *MYCN* amplification in NB-1, a *MYCN* gain in SH-SY5Y, a high-level *ALK* amplification in IMR-32 and *ALK* gains in BE(2)-C, Kelly, SH-SY5Y and LAN-6. *MYCN* and *ALK* status were also reliably determined from cell-free DNA derived from medium conditioned by the cell lines. *MYCN* and *ALK* copy numbers of subcutaneous neuroblastoma xenograft tumors were accurately determined from cell-free DNA in the mouse blood plasma. In a final validation step, we accurately distinguished *MYCN* and *ALK* copy numbers of the corresponding primary tumors using retrospectively collected blood plasma samples from 10 neuroblastoma patients. Our data justify the further development of molecular disease characterization using cell-free DNA in blood plasma from patients with neuroblastoma. This expanded molecular diagnostic palette may improve monitoring of disease progression including relapse and metastatic events as well as therapy success or failure in high-risk neuroblastoma patients.

## INTRODUCTION

Neuroblastoma, an embryonal tumor of neuroectodermal origin, accounts for 11% of all cancer-related deaths in children [1]. Molecular aspects create the extreme heterogeneity of this disease, spanning spontaneous regression to rapid metastasizing progression [2, 3]. Treatment scenarios range between observation only and multimodal concepts including high-dose chemotherapy with autologous stem cell rescue, surgery, radiotherapy and immunotherapy [1, 4]. Despite decades of considerable international efforts to improve outcome, long-term survival of high-risk disease remains as low as 40% [2, 4]. Two major remaining obstacles are managing resistance to induction therapy, which causes progression and early death, and managing chemotherapy-resistant relapses due to minimal residual disease, which can occur years after initial diagnosis. *MYCN* amplifications [5, 6] and activating *ALK* mutations or amplifications [7–10] define, among other molecular aberrations, patient subgroups with highly aggressive and frequently therapy-resistant neuroblastomas. One of the first targeted treatment options to become available for chemoresistant neuroblastomas is targeting activating *ALK* mutations or amplifications by blocking ALK tyrosine kinase activity [11–15]. Therapies indirectly targeting MYCN are not yet under clinical investigation. Promising preclinical strategies include binding or enzymatic inhibition of epigenetically acting proteins such as the BRD4 bromodomain protein [16, 17], the EZH2 [18] or DNMT1 [19] methyltransferases or the histone deacetylases [20, 21], and disturbing mechanisms maintaining MYCN protein stability via the inhibition of aurora kinase A (AURKA) [22].

OMICS-based investigations of the primary biopsy specimen cannot currently predict which tumors will develop resistance to first-line therapy, meaning that physicians have no molecular rationale for switching from an ineffective first-line therapy to a potentially life-saving second-line therapy without losing precious time. The invasive nature of surgical biopsies deters their sequential application to monitor disease in patients with cancer. Single biopsies often fail to reflect cancer dynamics, intratumor heterogeneity and drug sensitivities likely to change during cancer evolution and treatment. Emerging data indicate that implementing molecular characterization of tumor surrogates such as cell-free nucleic acids [23–29], exosomes [30], metabolites [31], circulating and disseminated tumor cells [32, 33] isolated from blood, bone marrow und urine will improve molecular disease assessment for treatment selection, patient monitoring and outcome prediction for cancer patients. Liquid biopsies could capture the molecular landscape of all tumor clones, and provide a method to follow clonal evolution in tumor subpopulations and treatment response in real time. We aimed to establish *MYCN* and *ALK* droplet digital PCR for the routine assessment of copy number status from sequential neuroblastoma blood and bone marrow samples to assist risk stratification and detection of cancer progression via *MYCN-* or *ALK*-dependent tumor-promoting subpopulations.

## RESULTS

### Droplet digital PCR supports quantifiable *MYCN* copy number measurement in mixed total DNA lysates from neuroblastoma cells

**Figure 1:**
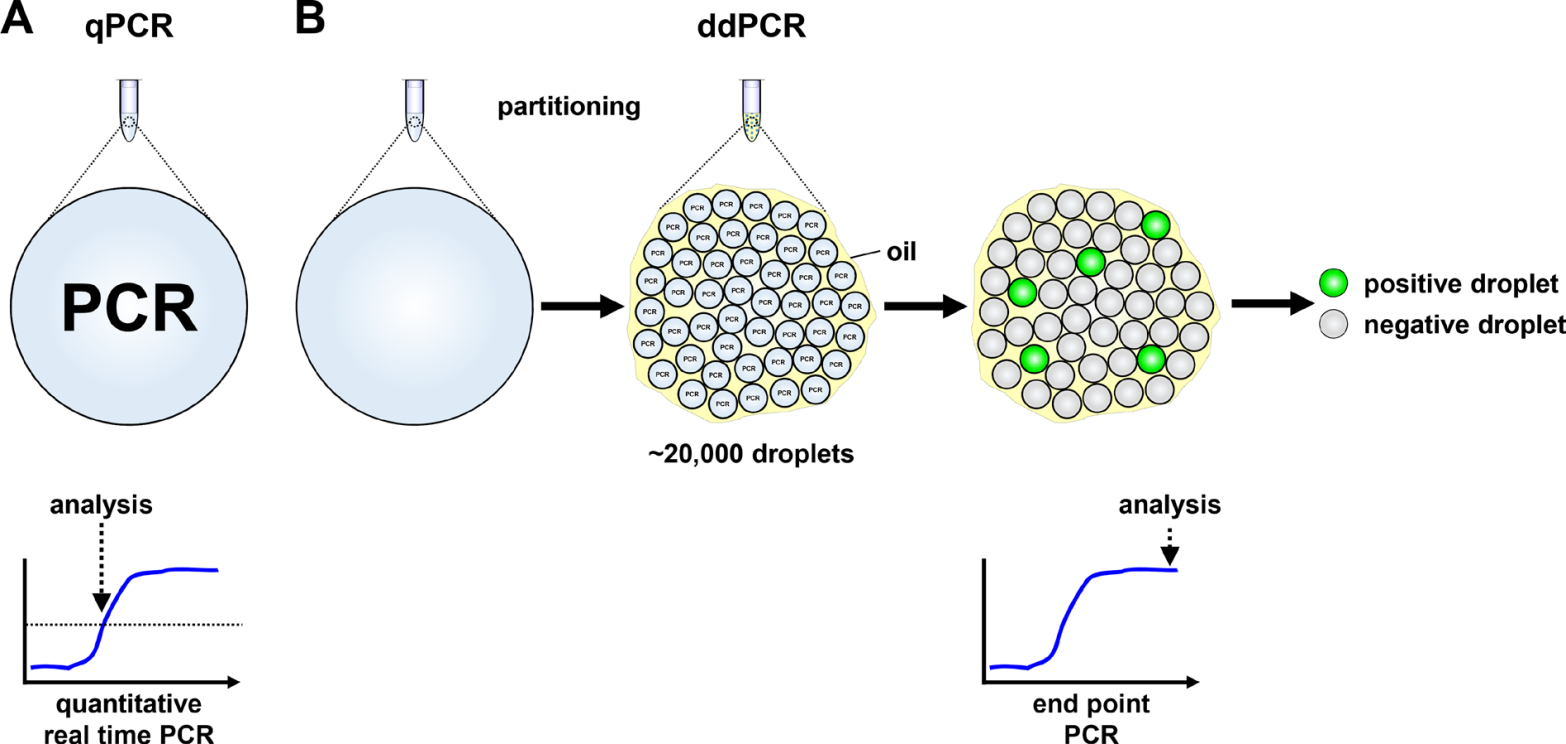
Schematic models of quantitative real-time and droplet digital PCR (**A**) Signal detection in quantitative real-time PCR (qPCR) is provided in a single reaction, measured in real-time and analyzed from the exponential phase of the reaction. (**B**) Droplet digital PCR (ddPCR) reaction reagents are partitioned into ∼20,000 droplets before PCR reactions proceed to the reaction plateau end point in individual droplets. Droplets are assessed as positive or negative from their fluorescent signal intensity.

**Figure 2:**
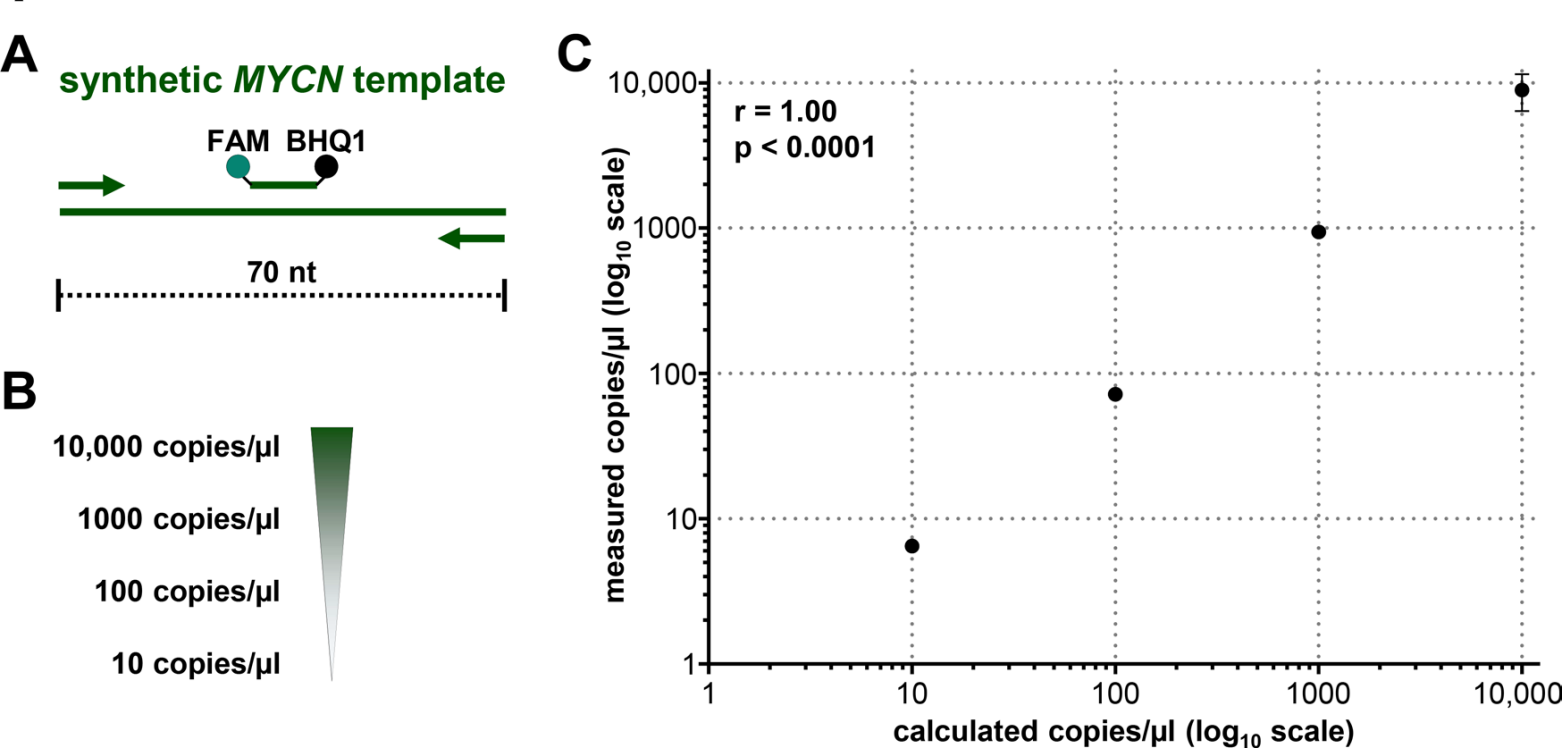
Droplet digital PCR maintains linearity over a broad copy number range (**A**) Schematic representation of the 70-nucleotide double-stranded synthetic *MYCN* template covering the complete sequence of the *MYCN* amplicon. Primers (arrows), probe (green line) and the positions of the FAM fluorescent dye (green dot) and BHQ1 quencher (black dot) are indicated. (**B**) Theoretically calculated numbers of *MYCN* copies after serial dilution of the synthetic template in H_2_O are shown. (**C**) Correlation analysis of theoretically calculated *MYCN* copies (x-axis) and copies of synthetic template assessed in dilution series using ddPCR (y-axis, as mean ± SD; *n* ≥ 3). Pearson’s correlation coefficient (r) and *p*-value is indicated.

**Figure 3:**
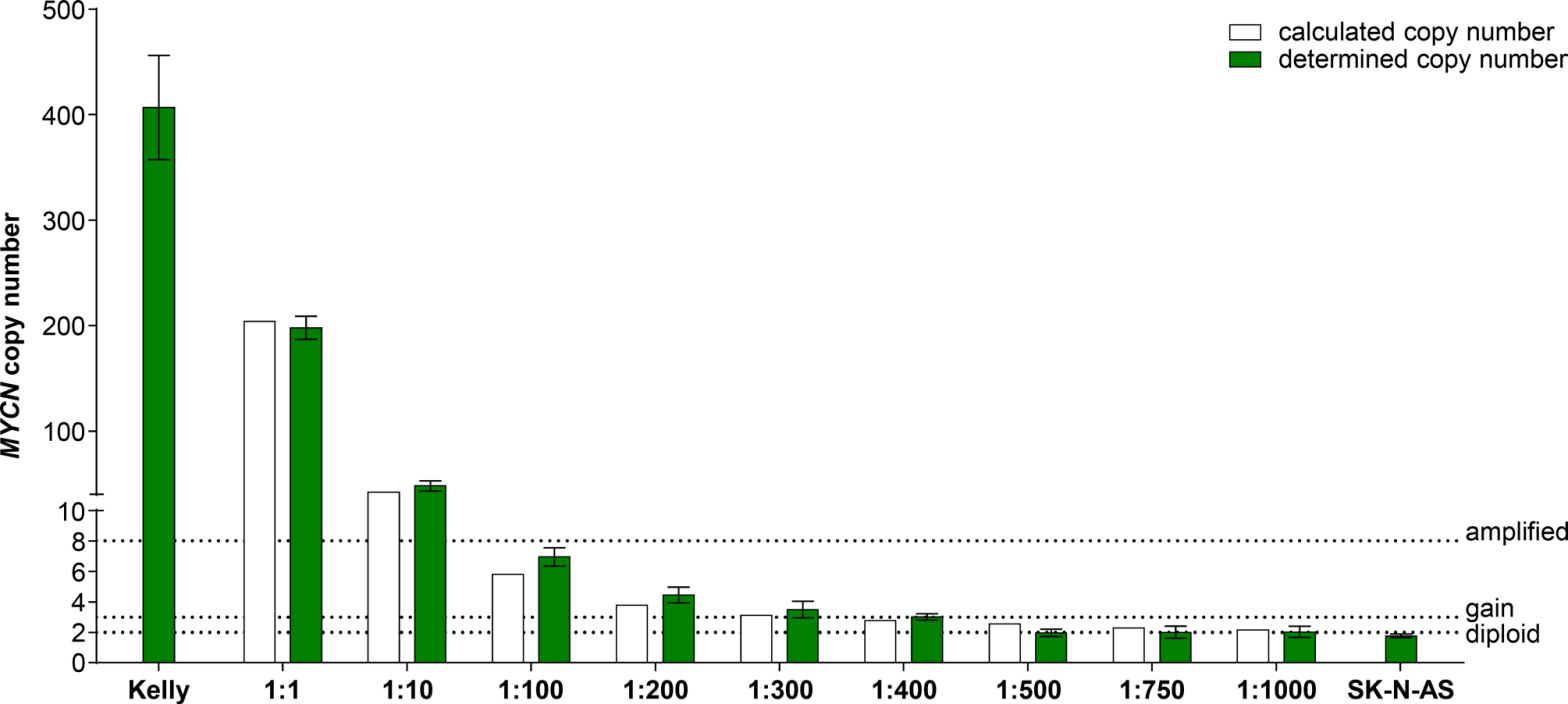
*MYCN* copy number detection in a titration series of mixed neuroblastoma cell lines either harboring or lacking *MYCN* amplification Kelly cells (*MYCN*-amplified) were titrated with SK-N-AS cells (diploid *MYCN*) to generate the indicated titration series. Genomic DNA was extracted and fragmented by sonication before *MYCN* copy number was assessed in 2 ng gDNA using ddPCR (green bars, mean ± SD; *n* ≥ 3). The number of *MYCN* copies calculated to be present at each titration step are shown by the white bars. Dashed lines indicate the thresholds used for FISH analysis of the neuroblastoma biopsy in the German NB2004 trial protocol: diploid *MYCN* (2 copies), *MYCN* gain (≥ 3 copies) and *MYCN* amplification (> 8 copies). Target gene copy number was analyzed using QuantaSoft analysis software (version 1.7.4, Bio-Rad). This software determined the copy number by calculating the ratio of the target molecule concentration A (copies/µl) to the reference molecule concentration B (copies/µl) times the number of reference species copies N_B_ in the human genome $\left(copy\;number=\frac{A}{B}\times N_{B}\right)$.

Droplet digital PCR (ddPCR) is a highly sensitive recently developed technology to quantify specific gene regions using a limiting dilution concept (Figure 1) [34, 35]. We set out to evaluate ddPCR for use with patient blood plasma samples and determine its accuracy and sensitivity for detecting neuroblastoma-specific *MYCN* copy number variation in cell-free DNA (cfDNA). A 70-nucleotide synthetic template and a convenient primer-probe set were designed for ddPCR-based *MYCN* detection (Figure 2A). We serially diluted the template to produce 10, 100, 1000 and 10,000 copies per µl H_2_O (Figure 2B). *MYCN* copy number was analyzed in the dilution series using ddPCR. The copy number detected by ddPCR perfectly correlated (Pearson’s correlation coefficient r = 1.00) with the theoretically calculated number of *MYCN* copies per µl H_2_O (Figure 2C). These data demonstrate that ddPCR detection maintains linearity within the range of 10 to 10,000 copies per µl in the absence of background molecules. We next assessed ddPCR sensitivity in detecting *MYCN* amplification in a mixture of genomic DNA isolated from two neuroblastoma cell lines. This experimental design was planned to reflect the status of a heterogeneous tumor containing cell clones with and without a *MYCN* amplification. We titrated the number of cells from the Kelly cell line, which harbors a *MYCN* amplification, with the number of SK-N-AS cells, which lack *MYCN* amplification, to generate a titration series comprising two cellular backgrounds. We then extracted genomic DNA (gDNA), sheared it by sonication and measured *MYCN* copy number by ddPCR. We detected 406.67 *MYCN* copies in undiluted Kelly cells and 1.76 *MYCN* copies in SK-N-AS cells (Figure 3). The *MYCN* copy number determined by ddPCR strongly correlated (Spearman’s correlation coefficient r = 0.93, *p* = 0.0007) with the *MYCN* copy number calculated from the gDNA titration series using the mixed cell background. The full extent of *MYCN* amplification present in the Kelly cells could not be detected in the normal diploid background of SK-N-AS cells if the gDNA mixture was diluted between 1:100 and 1:400, however, a *MYCN* gain was detected at these dilutions (Figure 3). Dilution to 1:500 no longer detected a *MYCN* gain caused by the presence of amplified cells in the normal diploid background using our ddPCR protocol. Our data demonstrate that ddPCR detection maintains linearity within the range of 10 to 10,000 copies per µl in the absence of background molecules and that ddPCR can be reliably used to detect *MYCN* amplification in gDNA from as little as one Kelly cell in a background of nine cells lacking *MYCN* amplifications.

### Droplet digital PCR yields exact *MYCN* and *ALK* copy numbers for 15 neuroblastoma cell lines

**Figure 4:**
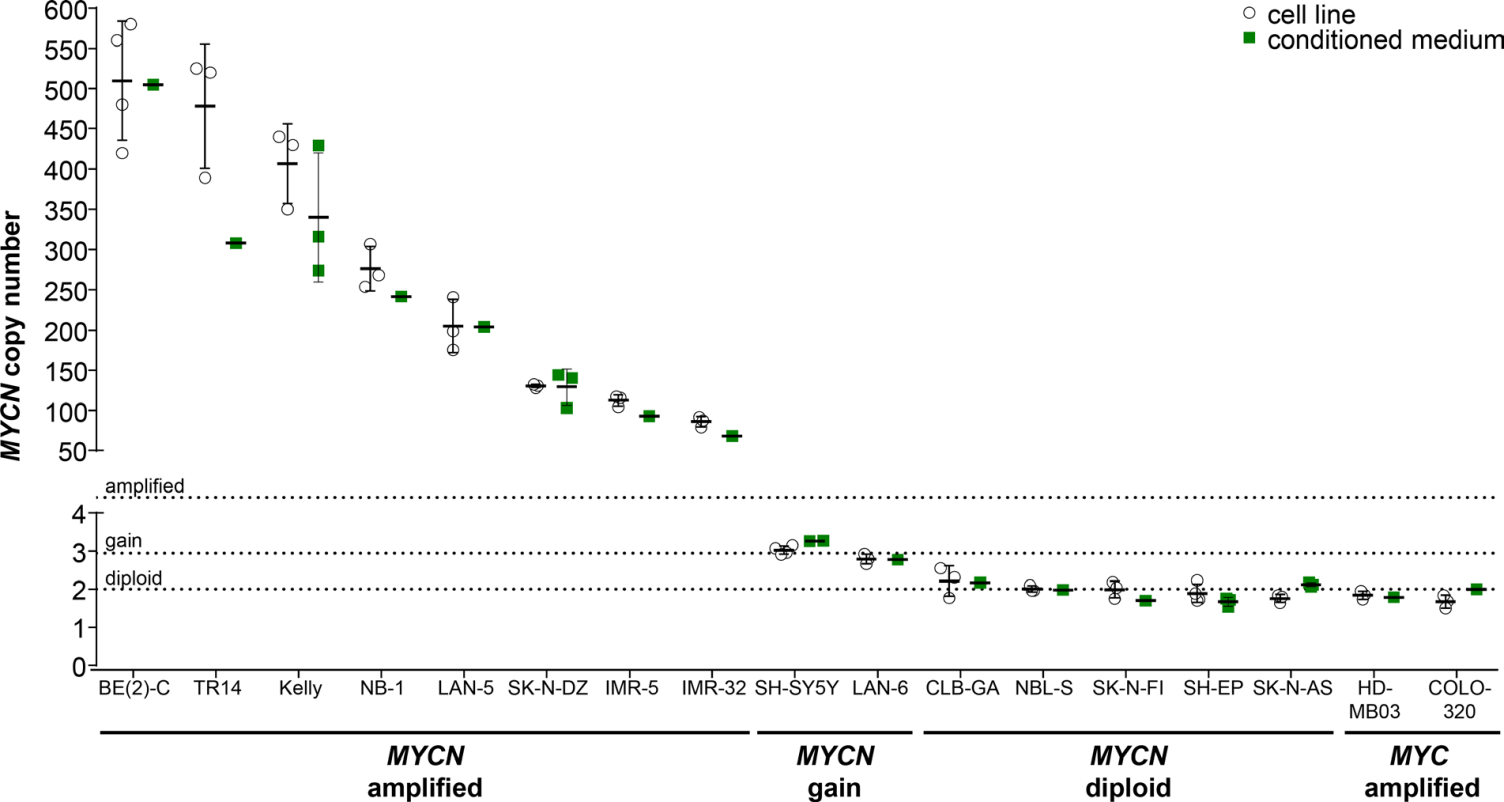
Comparison of absolute *MYCN* copy numbers determined by ddPCR for neuroblastoma cell lines Genomic DNA was extracted from cultured cells and fragmented by sonication before ddPCR for *MYCN* copy number (white circles, mean indicated by the line and SD indicated by the whiskers; *n* ≥ 3). Cell-free DNA was purified from medium conditioned by the cell lines, and *MYCN* copy number was determined by ddPCR (green squares, mean indicated by the line and SD indicated by the whiskers; *n* ≥ 1). The *MYC*-amplified HD-MB03 medulloblastoma [36] and COLO-320 colorectal carcinoma [5] cell lines were used as controls. Dashed lines indicate the thresholds used for FISH analysis of the neuroblastoma biopsy in the German NB2004 trial protocol: diploid *MYCN* (2 copies), *MYCN* gain (≥ 3 copies) and *MYCN* amplification (> 8 copies).

We selected 15 neuroblastoma cell lines previously reported to harbor *MYCN* and/or *ALK* amplifications, gains or the normal diploid chromosomal complements in the regions of *MYCN* and *ALK* as a panel in which to validate detection of gene amplification or gain by ddPCR. We included 1 medulloblastoma and 1 colon adenocarcinoma cell line, each reported to harbor a *MYC* amplification [36, 37], to test the specificity with which our ddPCR protocol detects *MYCN.* We extracted gDNA from this cell line panel, then sheared gDNA samples by sonication prior to ddPCR copy number detection. The German NB2004 trial protocol for FISH analysis of primary neuroblastoma samples designates *MYCN* amplification as the detection of > 8 *MYCN* copies, gain as detection of 3 to 8 copies and ‘single-copy’ as 2 copies. We designated *MYCN* and *ALK* amplification for our ddPCR analyses as the detection of ≥ 8.01 copies, gain as detection of 2.74 to 8.00 copies and normal diploid as detection of 1.50 to 2.73 copies. The thresholds for ddPCR were statistically determined from our cell line data set. *MYCN* copy number relative to *NAGK* copy number, as a normal diploid reference gene, was analyzed in gDNA isolated from cell lines and corresponding conditioned medium, using our established ddPCR protocol. We confirmed the amplified status of *MYCN* in BE(2)-C, TR14, Kelly, NB-1, LAN-5, SK-N-DZ, IMR-5 and IMR-32 reported in the literature. The *MYCN* copy numbers we determined (Figure 4, Table 1) from gDNA for BE(2)-C, Kelly, LAN-5 and IMR-32 were distinctly higher than the amplification levels reported in the literature using Southern blotting, FISH, competitive PCR, SNP oligonucleotide arrays or other methods (reported ranges summarized in Table 1). We determined 276.2 ± 27.7 *MYCN* copies in gDNA from the NB-1 cell line (Figure 4, Table 1) to ascertain its amplified status, which was unclear from previous FISH assessments reporting 2 or 20 *MYCN* copies (Table 1). We confirmed a *MYCN* gain in the SH-SY5Y cell line that was unclear from the literature (2 or 3 copies, Table 1) using ddPCR analysis of gDNA (Figure 4, Table 1). *MYCN* copy numbers determined using ddPCR in gDNA (Figure 4, Table 1) confirmed the gain in LAN-6 and normal diploid status in the SK-N-AS, SH-EP, SK-N-FI, NBL-S and CLB-GA neuroblastoma cell lines, which are reported in the literature (Table 1). We measured 1.85 ± 0.11 *MYCN* copies in gDNA from the HD-MB03 medulloblastoma cell line and 1.67 ± 0.17 *MYCN* copies in gDNA from the COLO-320 colon adenocarcinoma cell line (Figure 4, Table 1), confirming the specificity of our ddPCR assay for *MYCN*. In conclusion, we confirmed the *MYCN* copy number status reported for most neuroblastoma cell lines examined in our panel, and provide evidence for a *MYCN* gain in the SH-SY5Y cell line and a strong *MYCN* amplification in the NB-1 cell line.

**Table 1:** *MYCN* and *ALK* copy numbers determined by ddPCR for established cell lines from cellular genomic DNA and conditioned medium-derived cell-free DNA

	*MYCN* copy number^a^			*ALK* copy number^a^		
Cell line	genomic DNA	cell-free DNA	genomic DNA in literature^b^	genomic DNA	cell-free DNA	genomic DNA in literature^b^
BE(2)-C	510.0 ± 73.9	505.0	50^c^ –120^c [39, 66]^	4.28 ± 0.06	4.25	2 ^[77]^
TR14	478.0 ± 77.1	308.0	120 ^[41]^	1.93 ± 0.05	2.49	2-ampl. ^[12, 57]^
Kelly	406.7 ± 49.3	339.8 ± 80.4	100^c^ –240^c [38, 39, 66]^	3.05 ± 0.30	3.22 ± 0.17	2 ^[7, 77]^
NB-1	276.2 ± 27.7	241.5	2 –20 ^[8, 42]^	44.3 ± 2.4	56.7	30-40 ^[8, 15, 57]^
LAN-5	204.8 ± 33.0	204.0	50^c^ –150^c [38, 40]^	2.06 ± 0.07	2.26	2 ^[12, 57]^
SK-N-DZ	130.2 ± 2.0	129.0 ± 22.6	10^c [67]^	2.09 ± 0.09	2.25 ± 0.16	2 ^[7]^
IMR-5	112.3 ± 6.8	93.0	ampl. ^[68]^	132.8 ± 3.3	139.0	ampl. ^[7]^
IMR-32	86.0 ± 6.4	68.0	15^c^ –75^c [38, 40, 41, 66]^	102.8 ± 4.3	87.5	2-ampl. ^[12, 57, 76]^
SH-SY5Y	3.02 ± 0.11	3.27 ± 0.01	2–3 ^[69-72]^	3.04 ± 0.06	2.74 ± 0.08	2-3 ^[57, 71]^
LAN-6	2.79 ± 0.13	2.77	3 ^[73]^	3.01 ± 0.14	3.40	2 ^[57]^
CLB-GA	2.21 ± 0.40	2.17	2 ^[74]^	2.24 ± 0.12	2.09	2 ^[9, 57]^
NBL-S	2.01 ± 0.08	1.98	2 ^[75]^	2.07 ± 0.09	2.20	2 ^[57]^
SK-N-FI	1.99 ± 0.22	1.70	2 ^[75]^	2.15 ± 0.16	1.57	2 ^[12, 57]^
SH-EP	1.89 ± 0.24	1.67 ± 0.12	2 ^[39]^	1.97 ± 0.17	1.87 ± 0.18	2 ^[57]^
SK-N-AS	1.76 ± 0.11	2.12 ± 0.05	2 ^[39]^	1.92 ± 0.06	1.81 ± 0.05	2 ^[7, 12, 57]^
HD-MB03^d^	1.85 ± 0.11	1.79	2 ^[36]^	-	-	-
COLO-320^d^	1.67 ± 0.17	2.00	2 ^[5]^	-	-	-

^a^copy numbers as mean ± SD (genomic DNA, *n* ≥ 3 and cell-free DNA, *n* ≥ 1)

^b^For comparison, the *MYCN* and *ALK* values reported in the literature were included.

^c^amplification (fold)

^d^control cell lines lacking *MYCN* amplification and harboring *MYC* amplifications

**Figure 5:**
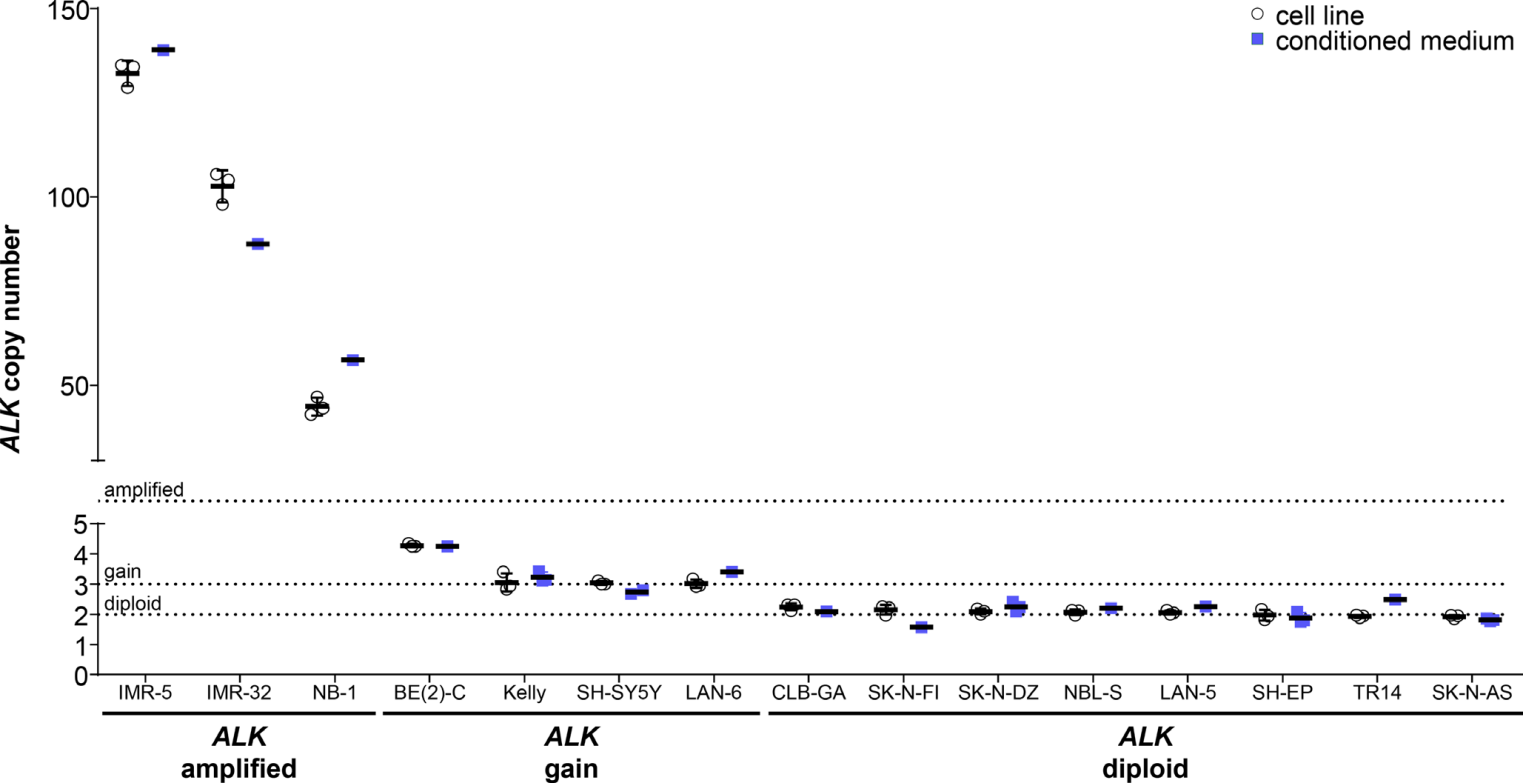
Comparison of absolute *ALK* copy numbers determined by ddPCR for neuroblastoma cell lines Genomic DNA was extracted from cultured cells and fragmented by sonication before *ALK* copy number was determined by ddPCR (white circles, mean indicated by the line and SD indicated by the whiskers; *n* = 3). Cell-free DNA was purified from medium conditioned by the cell lines, and copy numbers were detected by ddPCR (blue squares, mean indicated by the line and SD indicated by the whiskers; *n* ≥ 1). Dashed lines indicate thresholds for diploid (2 copies), gain (≥ 3 copies) and amplified (> 8 copies) status.

We analyzed *ALK* copy numbers relative to *NAGK* copy numbers using ddPCR of gDNA from our neuroblastoma cell line panel. We confirmed *ALK* amplifications in the IMR-32, NB-1 and IMR-5 neuroblastoma cell lines, which were previously reported in the literature (Figure 5, Table 1). Droplet digital PCR detected 102.8 ± 4.3 *ALK* copies in the IMR-32 cell line to confirm a high-level amplification, where multiplex ligation-dependent probe amplification had previously detected normal diploid status and analysis by SNP array or arrayCGH detected an amplification or partial amplification of unspecified copy number, respectively (Table 1). The *ALK* copy number determined using our protocol (Figure 5, Table 1) for NB-1 corresponded well to the amplification level assessed by Southern blotting (30– 40 copies) reported in the literature (Table 1). The level of *ALK* amplification has not been previously quantified for IMR-5 (Table 1), which we show harbors 132.8 ± 3.3 *ALK* copies (Figure 5, Table 1). *ALK* gains were detected in the BE(2)-C, Kelly, LAN-6 and SH-SY5Y cell lines using our ddPCR protocol (Figure 5, Table 1). Our data specifically confirm the *ALK* gain in SH-SY5Y that was unclear from the literature (2 or 3 copies, Table 1). In contrast, our ddPCR data contradicted previous reports of normal diploid *ALK* in the BE(2)-C, Kelly and LAN-6 cell lines (Table 1). We confirmed normal diploid status of *ALK* in the TR14 cell line (Figure 5, Table 1), which was unclear from previous studies reporting either amplified or normal diploid *ALK* status (Table 1). We also confirmed normal diploid *ALK* status (Figure 5, Table 1) in the SK-N-AS, SH-EP, LAN-5, NBL-S, SK-N-DZ, SK-N-FI and CLB-GA cell lines, which was previously reported (Table 1). We provide exact *ALK* copy number data for our neuroblastoma cell line panel that adds to previous reports classifying them only as diploid or amplified, and confirmed the reported *ALK* status with 6 exceptions. We provide evidence for the diploid status of *ALK* in the TR14 cell line, *ALK* gains in the BE(2)-C, Kelly, SH-SY5Y and LAN-6 cell lines and a strong *ALK* amplification in the IMR-32 cell line.

### *MYCN* and *ALK* copy number can be reliably determined from conditioned medium

**Figure 6:**
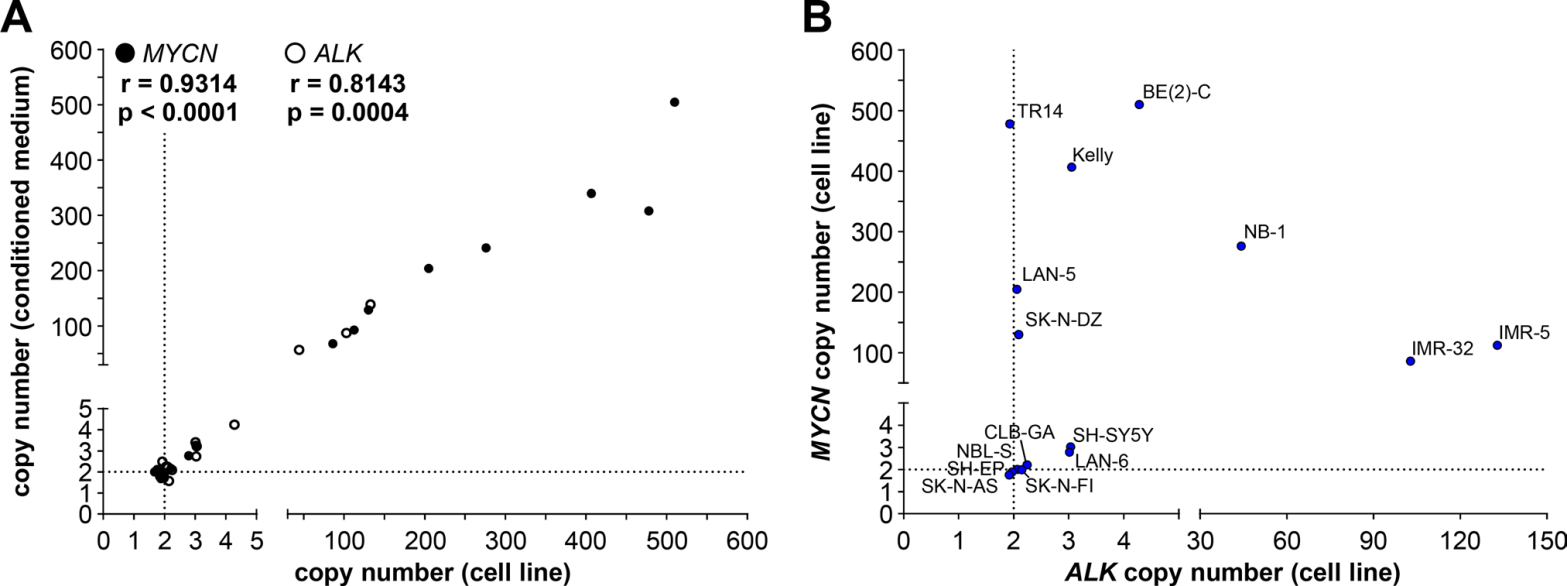
*MYCN* and *ALK* copy numbers determined by ddPCR of genomic DNA correlate with copy numbers determined from the corresponding cell-free DNA (**A**) Copy numbers determined by ddPCR from genomic DNA from each cell line is plotted on the x-axis and copy numbers determined by ddPCR from the corresponding cell-free DNA isolated from conditioned medium is plotted on the y-axis. Values presented in Figures 4 and 5 were used. Significance of the correlation between the 2 DNA sources for determining *MYCN* (black circles) and *ALK* (white circles) copy numbers was assessed from the plot, and the Spearman correlation coefficient (r) and *p*-value are indicated. Dashed lines indicate the position of diploid gene status. (**B**) Plotting *MYCN* copy numbers against corresponding *ALK* copy numbers divided neuroblastoma cell lines into different groups based on combined copy number status. *MYCN* copy numbers determined by ddPCR from genomic DNA from the indicated cell lines are plotted along the y-axis against *ALK* copy numbers determined by ddPCR from genomic DNA from the same cell lines (x-axis). Same values as those plotted in A are used. Dashed lines indicate the position of diploid gene status.

As an initial step towards copy number detection in acelluar biosamples such as blood plasma, we analyzed *MYCN* and *ALK* amplification status from cfDNA in medium conditioned by neuroblastoma, medulloblastoma and colon adenocarcinoma cell lines. The copy numbers detected using cfDNA in our ddPCR protocol were compared with copy numbers determined from cellular gDNA. We purified cfDNA from medium conditioned by our cell line panel. The *MYCN* amplifications we detected using ddPCR of gDNA in 8 neuroblastoma cell lines ranged from the 86.0 ± 6.4 *MYCN* copies measured in IMR-32 cells to the 510.0 ± 73.9 *MYCN* copies measured in BE(2)-C cells (Figure 4, Table 1), and all 8 amplifications could also be deduced from cfDNA. *MYCN* amplifications at the levels of 68 *MYCN* copies for IMR-32 and 505 *MYCN* copies for BE(2)-C were ascertained from cfDNA, which corresponded well with copy numbers determined from gDNA (Figure 4, Table 1). Absolute *MYCN* copy numbers determined from cfDNA and gDNA were identical for LAN-5 and SK-N-DZ. Absolute *MYCN* copy numbers determined for the TR14 cell line from cfDNA varied most strongly, with 308 copies detected in cfDNA and 478.0 ± 77.1 copies determined from cellular gDNA (Figure 4, Table 1). Our ddPCR assessment of cfDNA from the SH-SY5Y cell line also confirmed the *MYCN* gain detected in gDNA (Figure 4, Table 1). Likewise, the *MYCN* gain detected in the LAN-6 cell line was detectedin cfDNA (Figure 4, Table 1). The *MYCN* normal diploid status detected using gDNA was confirmed using cfDNA for the neuroblastoma cell lines, CLB-GA and SK-N-AS, as well as the *MYC*-amplified medulloblastoma and colon adenocarcinoma cell lines used as controls for *MYCN* assay specificity (Figure 4, Table 1). The *MYCN* copy numbers we measured in conditioned medium-derived cfDNA significantly correlated (Spearman’s correlation coefficient r = 0.9314) with the copy numbers detected in corresponding cellular gDNA for all 17 cell lines assessed (Figure 6A). Taken together, our data show that our ddPCR protocol can clearly distinguish between *MYCN* amplification, gain and normal diploid status in cell lines using medium-derived cfDNA.

We further validated copy number detection by ddPCR of cfDNA using our assay for *ALK* copy number in our neuroblastoma cell line panel. *ALK* copy numbers determined using cfDNA from the IMR-5, IMR-32 and NB-1 cell lines were close to the values determined from gDNA (Figure 5, Table 1), and showed that high-level *ALK* amplifications could also be detected using cfDNA. *ALK* copy numbers determined from cfDNA confirmed the *ALK* gains we detected in gDNA. Absolute *ALK* copy numbers determined from cfDNA from the BE(2)-C, Kelly, SH-SY5Y and LAN-6 cell lines corresponded well to those determined from gDNA (Figure 5, Table 1). *ALK* diploid status was also confirmed in all neuroblastoma cell lines using cfDNA. Absolute *ALK* copy numbers determined using cfDNA from the CLB-GA, SK-N-DZ, SH-EP and SK-N-AS cell lines corresponded well to those determined from gDNA (Figure 5, Table 1). *ALK* copy number determined for the TR14 cell line from cfDNA was slightly higher than the 1.93 ± 0.05 *ALK* copies determined from gDNA, but is still designated as normal diploid according to our ddPCR thresholds (Figure 5, Table 1). *ALK* copy numbers detected using cfDNA also significantly correlated (Spearman’s correlation coefficient r = 0.8143) with copy numbers measured in corresponding gDNA from all 15 neuroblastoma cell lines assessed (Figure 6A). These results further validate the accurate detection of gene copy number using cfDNA derived from conditioned medium for assessment of *ALK* amplification, gain and diploid status.

Because *MYCN* (2p24.3) and *ALK* (2p23.2-2p23.1) are both located on chromosome 2p, we assessed the extent of correlation between *MYCN* and *ALK* copy numbers. Plotting the *MYCN* copy numbers against corresponding *ALK* copy numbers divided our 15-cell line panel into 6 groups based on combined *MYCN* and *ALK* status (Figure 6B). One group contains neuroblastoma cell lines with normal diploid status of both genes (SK-N-AS, SH-EP, NBL-S, SK-N-FI and CLB-GA). The LAN-6 and SH-SY5Y cell lines belong to a second group harboring gains in both *MYCN* and *ALK*, indicating a larger region containing both genes may have been gained during a single event. *MYCN-*amplified cell lines were distributed within different groups according to their *ALK* status. The SK-N-DZ, LAN-5 and TR14 cell lines maintained their *ALK* diploid status in combination with *MYCN* amplification. The Kelly and BE(2)-C cell lines acquired *ALK* gains in addition to *MYCN* amplification, indicating separate events driving *MYCN* and *ALK* aberrations in these cell lines. The IMR-32 and IMR-5 cell lines harbor approximately equal *ALK* and *MYCN* copy numbers, and the NB-1 cell line harbors many more *MYCN* than *ALK* copies (Figure 6B, Table 1) indicating a single-event and multi-event mechanism creating the copy number landscape for *MYCN* and *ALK* in these 2 groups, respectively. Interestingly, no cell line harbored an *ALK* gain or amplification without a *MYCN* gain or amplification. Our ddPCR data from cell lines provide mechanistic insights into gain or amplification of regions on chromosome 2p in neuroblastoma.

### Plasma collected from mice harboring xenografts and from patients reflects tumor *MYCN* and *ALK* copy number status

**Figure 7:**
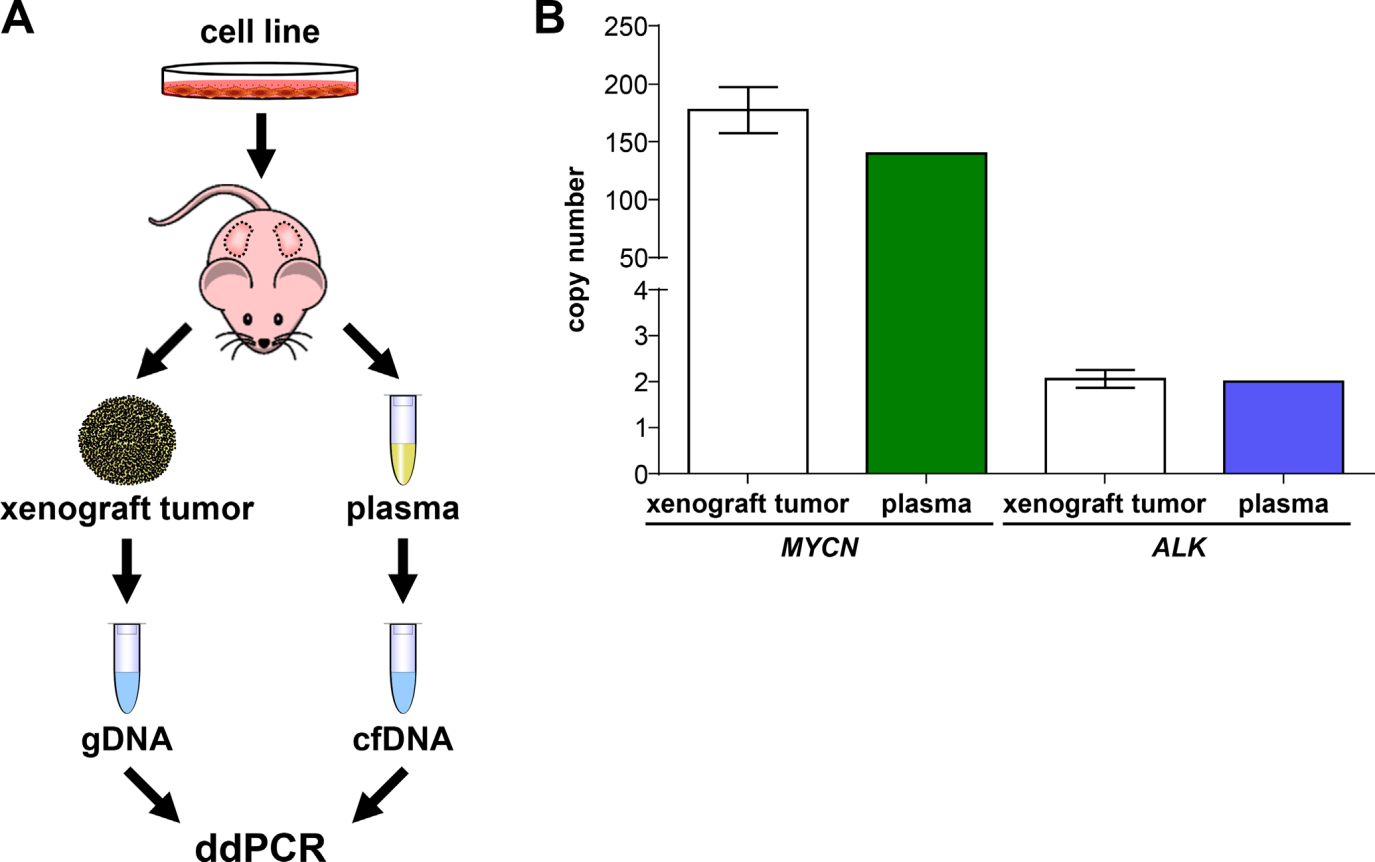
*MYCN* and *ALK* copy numbers assessed in cell-free DNA from plasma reflect copy numbers determined from xenograft tumor genomic DNA (**A**) Schematic workflow summarizing xenografting of the OHC-NB1 neuroblastoma cell line and sample preparation from genomic DNA (gDNA) and cell-free DNA (cfDNA) for droplet digital PCR (ddPCR). OHC-NB1 cells were subcutaneously injected into both flanks of 3 NMRI-Foxn1nu mice for gene copy number determination. Genomic DNA was extracted from each xenograft tumor and fragmented by sonication prior to ddPCR. Total blood plasma from the 3 mice was pooled for cfDNA isolation. (**B**) White bars show *MYCN* and *ALK* copy numbers detected by ddPCR using xenograft tumor genomic DNA (mean ± SD; *n* = 6) as the reference base. *MYCN* (green bar) and *ALK* (blue bar) copy numbers detected by ddPCR of cell-free DNA isolated from pooled mouse plasma (*n* = 1) are shown.

To initially test ddPCR detection capabilities in context with blood plasma, we compared *MYCN* and *ALK* copy numbers in xenograft tumors growing subcutaneously in mice with those measured in cfDNA isolated from mouse blood plasma, thereby avoiding the dilution effect potentially arising from damaged human white blood cells. Neuroblastoma cells were subcutaneously injected into both flanks of 3 NMRI-Foxn1nu mice for the analysis of *MYCN* and *ALK*, and the mouse cohorts monitored until the largest xenograft tumor reached a tumor volume of 1500 mm³ at day 40 (Figure 7A). Xenografts were established from a neuroblastoma cell line, named OHC-NB1 and established by our group (unpublished data), that harbors a high-level *MYCN* amplification and normal diploid *ALK*. As the positive control for tumor copy number, gDNA was extracted from each xenograft tumor and sonicated for ddPCR. Total blood plasma was collected from 3 mice by orbita puncture and pooled for cfDNA isolation. *MYCN* and *ALK* copy numbers were assessed in the cfDNA isolated from the plasma pooled using ddPCR. Our ddPCR protocol detected 139.75 *MYCN* copies in plasma-derived cfDNA, which was similar to the copy number (177.50 ± 19.81) determined from xenograft tumor gDNA (Figure 7B). *ALK* copy number was also assessed in both plasma-derived cfDNA and tumor gDNA using ddPCR. We detected 2.00 *ALK* copies using plasma-derived cfDNA, which was within the error range of the *ALK* copy number (2.06 ± 0.02) detected in the tumor gDNA. Our ddPCR protocol accurately detected the *ALK* normal diploid status and amplified *MYCN* status of the xenograft tumor from mouse blood plasma. These data demonstrate that cfDNA is released by subcutaneously grown human tumor cells into the mouse bloodstream in sufficient quantity to calculate tumor cell gene copy number from blood plasma, and confirm proof-of-principle for using our ddPCR protocol to detect *ALK* and *MYCN* copy numbers from patient blood plasma samples.

**Figure 8:**
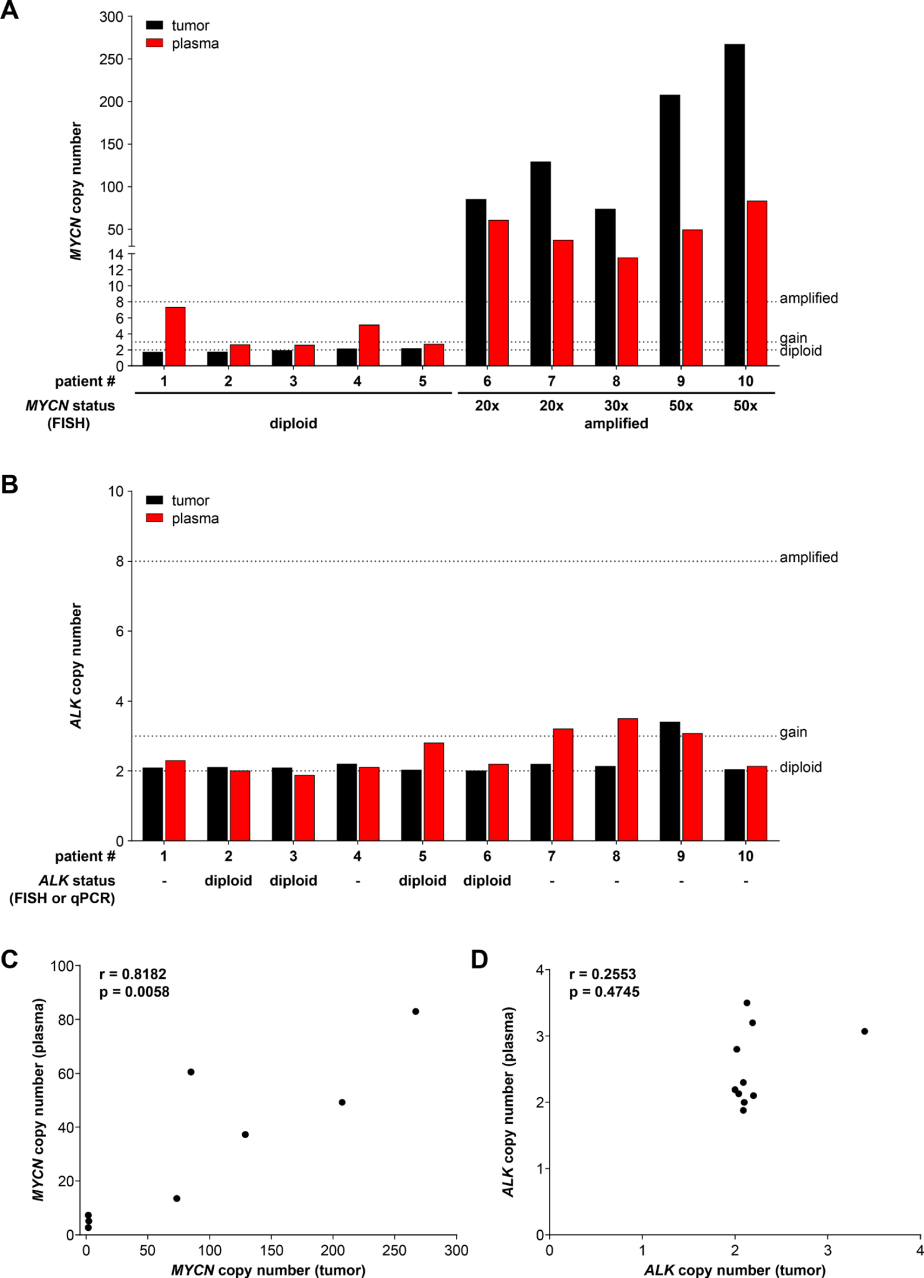
Droplet digital PCR accurately assesses *MYCN* and *ALK* copy numbers from neuroblastoma patient plasma and the corresponding primary tumor biopsy *MYCN* (**A**) and *ALK* (**B**) copy numbers were determined by ddPCR from retrospectively collected biomaterial samples from 10 neuroblastoma patients. Black bars show numbers determined from genomic DNA isolated from the tumor biopsy (single measurements). Red bars show values determined from cell-free DNA in the corresponding 200 µl plasma sample (single measurements). *MYCN* and *ALK* status determined at diagnosis (shown below bar graphs in A and B) were provided from patient records and were determined by FISH or qPCR (indicated) analysis. Dashed lines indicate the thresholds used for FISH analysis of the neuroblastoma biopsy in the German NB2004 trial protocol: diploid *MYCN* (2 copies), *MYCN* gain (≥ 3 copies) and *MYCN* amplification (> 8 copies). Correlation analysis of copy numbers determined from plasma-derived cell-free DNA (y-axis) and tumor biopsy genomic DNA (x-axis) is shown for *MYCN* (**C**) and *ALK* (**D**). Spearman correlation coefficients (r) and *p*-values are indicated.

We next evaluated ddPCR-based *MYCN* and *ALK* copy number assessment in blood plasma samples retrospectively collected at diagnosis from 10 neuroblastoma patients with known *MYCN* status. For comparison *MYCN* and *ALK* copy number assessment was performed in blood plasma samples collected from 16 healthy individuals. The German Neuroblastoma Biobank (Cologne) provided blood plasma samples paired with genomic DNA from the corresponding primary tumor from patients treated within the German NB2004 trial. Fragmentation of tumor gDNA was achieved by direct enzymatic digestion in the ddPCR reaction mixture due to the low DNA concentration in the 20 µl samples provided in water, and cfDNA was purified from the plasma samples before ddPCR. *MYCN* and *ALK* copy numbers were analyzed using our ddPCR protocol in the matched gDNA and cfDNA patient samples as well as in the cfDNA samples from healthy individuals. *MYCN* copy number in cfDNA samples from healthy individuals ranged between 1.54 to 2.60, and *ALK* copy number was between 1.64 and 2.50 (Table 3), indicating in line with the thresholds set a normal diploid status in all samples analyzed. FISH analysis conducted on the primary tumor for staging at diagnosis was used as the reference for *MYCN* status. We measured *MYCN* copy numbers in the range of 1.70 to 2.16 in tumor gDNA from patients 1–5 using ddPCR, which were in complete accordance with the diploid *MYCN* status determined by FISH (Figure 8A, Table 2). *MYCN* copy numbers ranging from 73.5 to 267.0 were detected using ddPCR of tumor gDNA from patients 6–10 (Figure 8A, Table 2), who were diagnosed with *MYCN*-amplified neuroblastomas according to FISH. *MYCN* copy numbers detected by ddPCR were higher than the amplification levels determined by FISH for each tumor. Copy numbers determined by ddPCR discriminated between a range of 73.5 to 129.0 for the 20- to 30-fold *MYCN* amplification levels determined by FISH and a range of 207.5 to 267.0 for the 50-fold *MYCN* amplification levels. *MYCN* copy numbers detected using plasma-derived cfDNA significantly correlated (Spearman’s correlation coefficient r = 0.8182) with the copy numbers measured in tumor gDNA (Figure 8C). We measured slightly higher *MYCN* copy numbers using plasma-derived cfDNA than the corresponding tumor gDNA for patients 2, 3 and 5 (Figure 8A, Table 2). *MYCN* copy numbers determined using plasma-derived cfDNA were distinctly higher than those determined from corresponding tumor gDNA for patients 1 and 4, respectively (Figure 8A, Table 2). *MYCN* copy numbers from plasma-derived cfDNA were consistently lower than the corresponding tumor gDNA from patients 6–10 with *MYCN*-amplified tumors (Table 2). *ALK* copy numbers determined using either plasma-derived cfDNA or tumor gDNA revealed normal diploid status in the samples from patients 1–4, 6 and 10 (Figure 8B, Table 2). Tumor gDNA from patients 5, 7 and 8 also revealed *ALK* diploid status, however, corresponding plasma-derived cfDNA indicated an *ALK* gain in these patients (Figure 8B, Table 2). An *ALK* gain of 3.40 and 3.07 copies was detected using tumor gDNA and plasma-derived cfDNA, respectively, from patient 9 (Figure 8B, Table 2). *ALK* gains were detected using plasma-derived cfDNA from 3 of 5 patients with *MYCN*-amplified tumors (patients 7, 8 and 9), but a possible *ALK* gain in a tumor with diploid *MYCN* status was only detected in patient 5 (Table 2). *ALK* copy numbers assessed using ddPCR of tumor gDNA from patients 2, 3, 5 and 6 were in complete accordance with the diploid *ALK* status determined by FISH or qPCR (Table 2). The copy numbers determined from plasma-derived cfDNA also confirmed the diploid *ALK* status in patients 2, 3 and 6, whereas plasma-derived cfDNA indicated an *ALK* gain in patient 5 (Figure 8B, Table 2). *ALK* copy numbers detected in plasma-derived cfDNA did not correlate (Spearman’s correlation coefficient r = 0.2553) with the copy number determined using tumor gDNA (Figure 8D). This is likely because of the narrow range of copy number (very near to 2 in most cases) values obtained for a small number of patients. These data indicate that our ddPCR protocol accurately assessed *ALK* copy numbers using plasma-derived cfDNA and tumor gDNA. Our ddPCR protocol enabled the detection of slight *ALK* copy number differences between cfDNA and gDNA. Our results for this 10-patient pilot cohort utilizing only archived tumor and blood plasma samples demonstrate that ddPCR analysis of either tumor gDNA or plasma-derived cfDNA accurately distinguished between *MYCN*-amplified and normal diploid status determined by FISH. Our ddPCR protocol required only 2 ng of tumor DNA, and assessed *MYCN* copy number more precisely than FISH over a wide copy number range. Plasma-derived cfDNA also produced *MYCN* copy numbers that correlated well with those determined from tumor gDNA.

**Table 2:** *MYCN* and *ALK* copy numbers determined by ddPCR from neuroblastoma patient blood plasma samples and paired genomic DNA from the corresponding primary tumor biopsy

	*MYCN* copy number			*ALK* copy number		
Patient	genomic DNA (tumor biopsy)	cell-free DNA (plasma)	reference value^a^ (tumor biopsy)	genomic DNA (tumor biopsy)	cell-free DNA (plasma)	reference value^b^ (tumor biopsy)
1	1.70	7.30	diploid	2.09	2.30	-
2	1.73	2.65	diploid	2.10	2.00	diploid
3	1.93	2.60	diploid	2.09	1.88	diploid
4	2.14	5.10	diploid	2.20	2.10	-
5	2.16	2.70	diploid	2.02	2.80	diploid
6	85.0	60.5	20x amplified	2.00	2.19	diploid
7	129.0	37.2	20x amplified	2.19	3.20	-
8	73.5	13.5	30x amplified	2.13	3.50	-
9	207.5	49.2	50x amplified	3.40	3.07	-
10	267.0	83.0	50x amplified	2.04	2.13	-

^a^FISH

^b^FISH or qPCR

**Table 3:** *MYCN* and *ALK* copy numbers determined by ddPCR in blood plasma samples from 16 healthy individuals

	*MYCN* copy number	*ALK* copy number
Healthy individual	cell-free DNA (plasma)	cell-free DNA (plasma)
1	1.73	1.93
2	2.40	1.72
3	1.79	2.10
4	1.72	2.16
5	1.59	2.17
6	1.90	2.10
7	1.77	1.84
8	2.23	1.97
9	1.80	2.22
10	2.60	2.40
11	1.90	2.00
12	1.54	1.72
13	1.70	2.50
14	2.00	1.74
15	1.70	1.70
16	2.06	1.64

## DISCUSSION

Here, we present ddPCR as an accurate method to assess *MYCN* and *ALK* copy numbers in tumor-derived cfDNA from blood plasma from neuroblastoma patients. The ddPCR method is highly sensitive and linear over a wide copy number range, and accurately discriminates between amplified, gain and normal diploid status. We validated our protocols in a panel of 15 neuroblastoma cell lines and 2 *MYC*-amplified non-neuroblastoma cell lines to control for assay specificity, and reveal evidence for different *MYCN* or *ALK* status than previously reported for 7 cell lines commonly used in research. Our ddPCR protocol yields absolute gene counts, making it easier to discriminate between gains and diploid status. We also validated our protocol in the mouse xenograft model, and demonstrate that *MYCN* and *ALK* copy numbers could be accurately determined from cfDNA in human blood plasma.

Gene copy number assessment using ddPCR is calculated through detection of the absolute concentration of target sequences and the diploid reference gene [34]. Droplet digital PCR has been used to assess gene copy numbers for cell lines and was compared to other methods used in published reports. *MET* copy number has been assessed in gDNA from gastric cancer and hepatocellular carcinoma cell lines and formalin-fixed, paraffin-embedded tumor samples from patient-derived xenograft models using ddPCR and SNP array or FISH [43]. The authors detected a significant correlation of copy numbers assessed using ddPCR and SNP arrays in cell lines or FISH in tumor samples. Belgrader and colleagues analyzed *HER2* copy numbers in gDNA from archived formalin-fixed, paraffin-embedded breast carcinoma samples using ddPCR or FISH [44]. They confirmed complete concordance of ddPCR and FISH for correct assessment of *HER2* amplifications. We demonstrated that ddPCR detection maintains linearity within a range of 10 to 10,000 copies per µl in dilution series using a synthetic *MYCN* template. These data confirm previous reports of ddPCR linearity within a range of 1 to ∼20,000 copies measuring *HER2* and reference gene *CEP17* in a dilution series of gDNA from the SK-BR-3 breast cancer cell line in H_2_O [45]. The concentration range of 10 to 10,000 copies per µl covered the absolute concentrations of each target sequence assessed for *MYCN*, *ALK* and *NAGK* in our cell line panel analyses, indicating the reliability of the copy number calculations.

We assessed or re-assessed *MYCN* and *ALK* copy numbers in gDNA from a panel of 15 cell lines commonly used in neuroblastoma research during the development and validation of our ddPCR protocol. We provide exact *ALK* copy numbers for our neuroblastoma cell line panel and our protocol confirmed the amplified, gain or diploid status in most cell lines. While our protocol confirmed the amplified, gain or diploid status of *MYCN* in most of the cell lines, absolute copy numbers determined by ddPCR were higher than reported values determined by different methods. Published estimates of *MYCN* amplification also varied in reports using the same or different methods. We determined 406.7 *MYCN* copies in gDNA from the Kelly cell line. A range of 100 – 120 *MYCN* copies were reported for the Kelly cell line using Southern blotting [38], while using competitive PCR detected 240 *MYCN* copies [39]. The IMR-32 cell line was reported to harbor a 15-25-fold *MYCN* amplification using Southern blotting [38], 25–75 *MYCN* copies using Southern blotting [40] or 50 *MYCN* copies using a SNP oligonucleotide array [41]. We assessed 86.0 *MYCN* copies in IMR-32 using ddPCR. The NB-1 cell line was reported as diploid for *MYCN* [8] or as *MYCN*-amplified with 20 copies estimated by FISH [42]. We confirmed *MYCN* amplification in the NB-1 cell line, which we show harbors 276.2 *MYCN* copies. The absolute quantitation of the *NAGK* reference gene provides the normal input DNA level and functioned as a control in our analyses. We avoided continuous culture of cell lines to reduce the risk of genomic alterations during maintenance, and think it is unlikely that the higher copy numbers are produced by additional chromosomal alterations acquired during culture. These data indicate that ddPCR can be reliably used as an accurate tool to assess copy number over a broad range as shown by our reported values for *MYCN* and *ALK* in 15 established neuroblastoma cell lines.

Single tumor biopsies do not adequately reflect clonal heterogeneity within the primary tumor (intratumoral heterogeneity), heterogeneity between different tumors or metastases in the same patient or changes in the tumor or metastases occurring during the course of treatment or progression. Spatial and temporal heterogeneity have been described for several cancer entities, including colorectal cancer [46], breast cancer [47], glioblastoma [48] and neuroblastoma [49–51]. We assessed *MYCN* and *ALK* copy numbers from archived single tumor biopsies from the 10-patient pilot cohort in this study as the control measure for copy number detection using the paired plasma samples. For some patients, tumor gDNA and plasma-derived cfDNA yielded different gene copy numbers. Copy numbers assessed using cfDNA indicated a gain for *MYCN* in 2 patients (1 and 4) and for *ALK* in 3 patients (5, 7 and 8), whereas corresponding tumor gDNA revealed normal diploid status. The higher copy numbers detected in tumor-derived circulating cfDNA may indicate more aggressive tumor clones either in the primary tumor or at metastatic sites that were not sampled by the single primary tumor biopsy. Large-scale longitudinal studies monitoring *MYCN* and *ALK* copy number status in cfDNA from individual patients and comparing these findings with the in-depth analysis of all tumor specimens collected from the respective patients are required to ultimately define the potential of cfDNA analysis in context with the identification and characterization of tumor heterogeneity in metastatic neuroblastoma disease. In all patients with a *MYCN*-amplified neuroblastoma, *MYCN* copy numbers determined using cfDNA were lower than those determined from tumor gDNA. The lower copy numbers detected in tumor-derived circulating cfDNA could be due to the dilution effect by damaged white blood cells generated through the suboptimal pre-analytic handling of the respective blood samples, which were as stated elsewhere originally not collected for cfDNA studies. Alternatively, this finding could reflect clonal tumor heterogeneity in the primary tumor, and be an average of the biopsied amplified clonal region and other non-amplified regions. Again, large-scale trials monitoring disease status by optimally collecting and analyzing blood and tumor tissue samples are needed to ultimately answer this question. The potential for detection of tumor heterogeneity using cfDNA has been suggested in several reports. Chicard and colleagues compared tumor genome copy number profiling of cfDNA using the OncoScan array and array CGH analysis of the primary neuroblastoma biopsy [51]. They identified *TERT* alterations, which are associated with aggressive high-risk neuroblastoma [52, 53], only in tumor-derived cfDNA (not tumor biopsy) from 2 patients. The authors hypothesized that cfDNA might reflect genetic alterations of more aggressive cell clones [51]. Our detection of *MYCN* gains in patients 1 and 4, and *ALK* gains in patients 5, 7 and 8 in plasma compared to the primary tumor biopsy also indicated these patients have subclonal amplifications in the primary tumor, which were not at the site of biopsy or present in distant undetected metastases. Sequence analysis of plasma-derived cfDNA detected all mutations detected in the primary tumor and/or its synchronous liver metastasis in a massive parallel sequencing study of samples from a patient with breast carcinoma [54]. Similarly, Chan and colleagues detected a composite pattern of specific copy number aberrations and mutations in plasma-derived cfDNA from a patient that corresponded to the breast carcinoma and 2 ovarian tumors synchronously present using massive parallel sequencing [55]. These data, although incidental, strongly support that differing copy numbers in cfDNA and tumor gDNA indicate the presence of tumor cell populations in the patient that are not captured by the single primary tumor biopsy. We conclude that this interpretation applies without restriction for the *MYCN* gains detected in plasma from patients 1 and 4, and the *ALK* gains detected in plasma from patients 5, 7 and 8 in our cohort. Even though our pilot cohort only includes 10 patients, it is astounding to speculate that single primary tumor biopsies may be underestimating the real tumor cell population in half of high-risk neuroblasoma patients.

Since retrospectively collected patient samples were analyzed in this study, blood was not sampled according to a standardized protocol for cfDNA detection. Blood samples may have spent longer than a day at room temperature before plasma preparation and storage at -80°C. Lysis of white blood cells before cfDNA preparation can dilute the tumor-derived DNA with normal cellular DNA. This dilution effect was previously demonstrated by titrating serum samples with increasing numbers of white blood cells, which masked high *MYCN*/*NAGK* ratios and prevented detection of *MYCN* amplification above a threshold [24]. We also simulated a dilution effect by titrating *MYCN*-amplified Kelly cells with SK-N-AS cells harboring the normal diploid *MYCN* complement, and showed that detection of the *MYCN* amplification was masked above a threshold of dilution with 99 normal diploid cells. This effect depends on both amplification level and degree of dilution. Visible hemolysis had occurred in plasma samples from 4 neuroblastoma patients (2, 6, 8 and 10), indicating that white blood cells may have also lysed in these samples. The lower copy numbers detected in plasma samples from patients with *MYCN*-amplified tumors could have resulted from dilution with gDNA from lysed normal blood cells. Although, we did not detect copy numbers closer to the tumor biopsy in the two plasma samples that lacked visible hemolysis (patients 7 and 9). Dilution may have reduced the copy numbers determined from all our plasma samples, which were collected under suboptimal conditions for cfDNA detection. These data emphasize the importance of a standardized protocol for blood sampling and plasma preparation (limiting time at room temperature) in the development of cfDNA-based diagnostic protocols for clinical application.

A review of the literature indicates that assessment of *MYCN* status using quantitative real-time PCR and serum/plasma-derived cfDNA presents a promising method to analyze neuroblastoma patient samples. Serum-based *MYCN* analysis has been shown capable of separating patients into groups with amplified and non-amplified tumors [24, 26]. Assay sensitivity was dependent on neuroblastoma stage, and was highest in metastasized disease [25]. *MYCN* copy number detection in tumor-derived cfDNA from plasma samples taken before and after surgery has been suggested as a useful evaluation step for surgery and neoadjuvant chemotherapy [26]. Kurihara and colleagues analyzed plasma-derived cfDNA from patients with a variety of childhood solid tumors before and after surgery using next-generation sequencing and ddPCR for mutations, deletions or amplifications, including *MYCN* status in neuroblastomas [27]. They confirmed the potential of cfDNA screening using next-generation sequencing and ddPCR for biomarker detection. Here, we also accurately distinguished between *MYCN-*amplified and *MYCN* diploid status in tumors using plasma-derived cfDNA. Taken together, these results justify molecular disease characterization using tumor-derived cfDNA in blood for longitudinal monitoring of patients with neuroblastoma.

A major challenge for tumor cfDNA analysis is the extremely low amount of circulating cfDNA present in blood in at least some patients [56]. Higher sensitivity and a requirement for lower absolute cfDNA amounts has been demonstrated for ddPCR in comparison with next-generation sequencing [56]. We have pursued ddPCR as the more suitable technology for repeated target gene copy number assessement in longitudinal measurements. *ALK* amplification is reported in 2–5% of primary neuroblastomas [7, 57–59]. It occurs almost exclusively with *MYCN* amplification, and has been detected in 7–15% of *MYCN*-amplified neuroblastomas [10, 60]. *ALK* gain was reported to occur in ∼15–20% of primary neuroblastomas [57, 58]. *ALK* copy numbers detected by ddPCR of plasma-derived cfDNA have not yet been reported for patients with neuroblastomas. We accurately assessed *ALK* copy numbers in the primary neuroblastomas from tumor-derived cfDNA in blood plasma samples. We detected *ALK* gains in 4 plasma samples, one of which was confirmed in the corresponding tumor sample. Contradictory reports of the association of *ALK* amplification or gain with survival have been published. *ALK* amplification is strongly associated with high-risk neuroblastoma and an inferior outcome, and multivariate analysis suggested an independent influence on survival for both *ALK* gain and amplification in a cohort of 1596 neuroblastoma samples enrolled in a COG biology study [61]. In contrast, *ALK* amplification was not a statistically independent marker for survival in models with *MYCN*, stage and age in a meta-analysis, combining a new series of 254 neuroblastomas and 455 published cases from 3 other cohorts [57]. Wang and colleagues identified only 3 neuroblastomas harboring *ALK* amplifications, all also harboring *MYCN* amplifications, in a cohort of 188 primary neuroblastomas from a patient cohort treated at the Beijing Children’s Hospital [62]. *ALK* amplification correlated with a decreased overall survival, whereas interestingly, *MYCN* or *ALK* gain were associated with a better prognosis. Thus, a *MYCN*-independent association of *ALK* amplification with outcome remains open. The authors concluded that classification of *MYCN* and *ALK* gene copy numbers can provide a powerful prognostic indicator. We theorize that the combined assessment of *MYCN* and *ALK* copy numbers using ddPCR of plasma-derived cfDNA during treatment and follow-up will improve risk assessment for patients and enable an early detection of relapse and metastasis. *MYCN* and *ALK* copy number assessment from plasma at diagnosis could also identify patients with possible subclonal amplifications, and this analysis should accompany tumor biopsy analyses to test its clinical predictive power for possible future use in treatment stratification.

This study presents ddPCR as an accurate tool to assess *MYCN* and *ALK* copy numbers in neuroblastoma cell lines, tumor samples and tumor-derived cfDNA in blood plasma. Our validation experiments in retrospective patient samples support the hypothesis that using tumor-derived cfDNA from patient plasma can improve detection of high-risk tumor subpopulations harboring genomic aberrations relevant for therapy choice and risk assessment. Analyzing cfDNA in liquid biopsies will enable longitudinal measurements for a patient, starting prior to therapy and continuing through treatment course and follow-up, and supporting multiple diagnostic and therapeutic decisions during all phases of care for patients with high-risk neuroblastoma. This approach may improve detection of *MYCN* and *ALK* subclonal amplifications, monitoring of disease progression and treatment response as well as relapse detection.

## MATERIALS AND METHODS

### Patient samples

Matched blood plasma and tumor tissue genomic DNA from primary neuroblastoma biopsies were obtained from the German Neuroblastoma Biobank (Cologne). All 10 neuroblastoma patients were registered in the German NB2004 clinical trial, and informed patient/parent consent was obtained during trial participation. The following preparatory or storage steps occurred prior to delivery of patient biosample residues for this study. Peripheral blood was stored for a minimum of 24 h, then centrifuged at approximately 1000 x *g* for 10 min before storage of the plasma at –80°C. The extracted tumor gDNA was stored at –80°C. Values for *MYCN* and *ALK* status determined within the NB2004 study protocol and maintained with patient records in the central trial database were communicated with the patient biomaterial samples. For comparison, blood plasma from 16 healthy individuals was collected.

### Animal experiments

Subcutaneous xenografts were created by injecting 10 × 10^6^ OHC-NB1 cells (unpublished data) suspended in 200 μl Matrigel (BD Biosciences, Heidelberg, Germany) into each flank of 3 NMRI-Foxn1nu mice. Tumor size was measured daily with a caliper, and volume was calculated by π/6(width×height×depth). Animals were sacrificed 40 days after grafting, and total blood from orbita puncture in the 3 mice was collected in a single EDTA tube and centrifuged 7 min at 3000 x *g*. Plasma was centrifuged 10 min at 3900 x *g* and the supernatant was stored at –80°C before sample preparation. Genomic DNA was extracted from the 6 tumors separately as reference tumor DNA. Animal handling and care conformed to national and EU regulatory standards of the Research Institutes for Experimental Medicine, *Charité - Universitätsmedizin Berlin* and experiments were approved by *Landesamt für Gesundheit und Soziales Berlin*.

### Cell culture

The BE(2)-C cell line was obtained from ECACC (Salisbury, UK) and the COLO-320, IMR-32, Kelly and SH-SY5Y cell lines from the DSMZ (Braunschweig, Germany). CLB-GA, IMR-5, LAN-5, LAN-6, NBL-S, SK-N-FI and TR14 were kindly provided by J.H. Schulte (Charité, Universitätsmedizin Berlin, Germany), NB-1 by I. Oehme (DKFZ, Heidelberg, Germany), SH-EP and SK-N-AS by L. Savelyeva (DKFZ, Heidelberg), and SK-N-DZ by A. Künkele (Charité, Universitätsmedizin Berlin). OHC-NB1 (unpublished data) and HD-MB03 [36] were established in the Deubzer laboratory. Cell line authenticity was validated by high-throughput SNP-based assays [63]. All cell lines were cultured in full media (described in Supplementary Methods) at 37°C, 5% CO_2_, and continuous culture was avoided to maintain low passage numbers and reduce the risk of genomic alterations occurring. Cells for experiments were grown in short-term culture from low-passage stock aliquots maintained in liquid nitrogen. All cell lines were regularly monitored for *Acholeplasma laidlawii* and other species of mycoplasma as well as squirrel monkey retrovirus (SMRV) infections using high-throughput, multiplexed testing [64].

### Genomic and cell-free DNA isolation

Genomic DNA was extracted from cell lines or xenograft tumors using the QIAamp DNA Mini kit (Qiagen, Hilden, Germany) according to the manufacturer’s instructions. Conditioned medium (2 ml) was collected, centrifuged at 2000 × *g* for 5 min and the supernatant stored at –80°C. Thawed conditioned media and plasma samples were centrifuged at 2000 × *g* for 5 min to clear debris, then supernatant was centrifuged at 20,000 × *g* for 5 min. Cell-free DNA was purified from processed conditioned media, 200 µl stored human patient plasma, 1000 µl stored healthy individual plasma and 600 µl mouse plasma using the QIAamp Circulating Nucleic Acid kit (Qiagen) then concentrated to 50 µl using the DNA Clean and Concentrator-5 kit (Zymo Research, Freiburg, Germany), both according to manufacturers’ directions. Neuroblastoma gDNA was extracted using the Qiagen Puregene Core kit A (Qiagen) according to the manufacturer’s instructions. Extracted DNA samples were quantified on a Qubit 2.0 fluorometer (Life technologies, Darmstadt, Germany). Purified genomic and cell-free DNA were stored at –80°C until *MYCN* and *ALK* copy number detection.

### Droplet digital PCR

Genomic DNA from cell lines and xenograft tumors was fragmented by sonication before ddPCR. Sonication of gDNA from primary neuroblastoma samples was not possible because of the small sample volumes (20 µl), so fragmentation was achieved by adding 5 U of AluI restriction enzyme (New England Biolabs, Frankfurt/Main, Germany) to each ddPCR reaction. The QX200 Droplet Digital PCR System (Bio-Rad Laboratories, Munich, Germany) was used to simultaneously detect *MYCN* (2p24.3) or *ALK* (2p23.2-2p23.1) in a duplex reaction with the normal diploid reference gene, N-acetylglucosamine kinase (*NAGK*, 2p13.3) (2-D plots of the duplex reactions shown in Supplementary Figure 1). TaqMan ddPCR duplex reaction mixtures included 2x ddPCR *Supermix for Probes (no dUTP)* (Bio-Rad Laboratories), each primer at final concentrations of 900 nM and each probe at final concentrations of 250 nM in a total volume of 20 µl. The primers and probes used for *MYCN* and *NAGK* ddPCR were previously described [24], and are listed in Supplementary Table 1. The fluorescence dyes and quenchers of the *MYCN* and *NAGK* probes were adapted for ddPCR. *ALK* primers were designed from the amplicon described by Bavi et al. [65] and the *ALK* probe was designed with Primer3 software (http://bioinfo.ut.ee/primer3-0.4.0/primer3/), and are listed in Supplementary Table 1. Reaction mixtures were loaded into droplet generator cartridges (Bio-Rad) together with 70 µl Droplet Generation Oil (Bio-Rad). Droplets were generated in the QX200 Droplet generator, and manually transferred into a 96-well PCR plate (Eppendorf, Hamburg, Germany) according to manufacturer’s recommendations. The PCR plate was heat-sealed with the PX1 Plate Sealer (Bio-Rad), and PCR reactions were performed on a T100 Thermo Cycler (Bio-Rad) with the following program: 1 cycle at 95°C for 10 min, 40 cycles at 94°C for 30 seconds and at 58°C for 1 minute and 1 cycle at 98°C for 10 min. After PCR amplification, droplets were measured in the QX200 ddPCR Droplet Reader, and target gene copy number was analyzed using QuantaSoft analysis software (version 1.7.4, Bio-Rad). This software determined the copy number by calculating the ratio of the target molecule concentration A (copies/µl) to the reference molecule concentration B (copies/µl) times the number of reference species copies N_B_ in the human genome $\left(copy\;number=\frac{A}{B}\times N_{B}\right)$. The double-stranded synthetic *MYCN* template for initial ddPCR testing was obtained from Metabion (Planegg, Germany) and had the sequence: 5′- GTGCTCTCCAATTCTCGCCTTCACTAAAGTTCCTTCCACCCTCTCCTGGGGAGCCCTCCTCTAGGCCATC-3′.

### Statistical analysis

Correlation analyses were performed using GraphPad Prism (version 6.00). *P*-values below 0.05 were considered significant.

## SUPPLEMENTARY MATERIALS FIGURE AND TABLE


