# Supplementary material for: Using droplet digital PCR to analyze MYCN and ALK copy number in plasma from patients with neuroblastoma
Source: Oncotarget. 2017 Jul 7;8(49):85234–51. doi: 10.18632/oncotarget.19076 (PMC5689606; doi:10.18632/oncotarget.19076)
Supplement: Supplementary file 1 [file oncotarget-08-85234-s001.pdf]

Using droplet digital PCR to analyze *MYCN* and *ALK* copy number in plasma from patients with neuroblastoma

SUPPLEMENTARY MATERIALS

MATERIALS AND METHODS

Cell culture

CLB-GA and TR14 were maintained in Dulbecco's modified Eagle medium (DMEM, Gibco, Paisley, UK) supplemented with 10% fetal calf serum (FCS, Biochrom, Berlin, Germany). BE(2)-C, IMR-32, SH-SY5Y and SK-N-AS were cultured in DMEM supplemented with 10% FCS and 1% non-essential amino acids (NEAA, Lonza, Cologne, Germany). SK-N-DZ were maintained in DMEM supplemented with 10% FCS and 1% L-glutamine (Gibco). LAN-6 were cultured in DMEM supplemented with only 20% FCS. COLO-320, LAN-5, NBL-S and SK-N-FI were maintained in Roswell Park Memorial Institute (RPMI) 1640 medium (Gibco) supplemented only with 10% FCS, and 1% NEAA was also added for the culture of HD-MB03, Kelly, NB-1 and SH-EP. IMR-5 was cultured in RPMI 1640 supplemented with 10% FCS, 1% NEAA and 1% L-glutamine. The OHC-NB1 cell line was grown in DMEM supplemented with 10% FCS, 1% NEAA, 20 ng/ml EGF (Promocell, Heidelberg, Germany) and 20 ng/ml bFGF (Promocell).

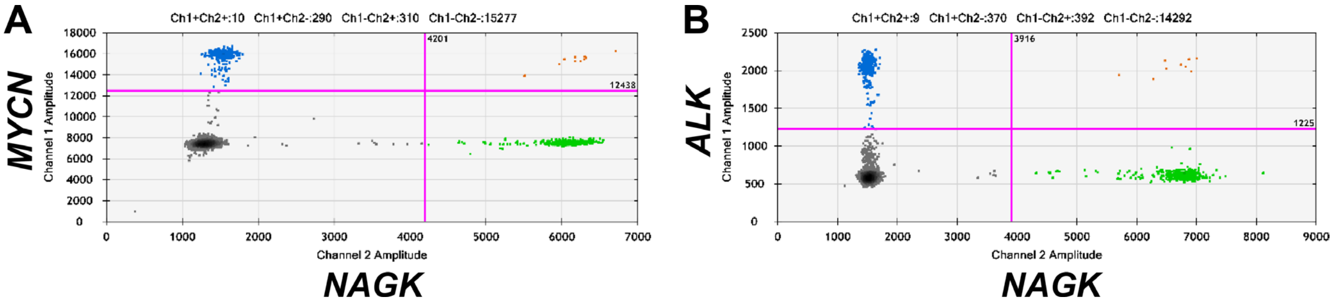

Supplementary Figure 1: Exemplary 2-D plots of droplet fluorescence in the ddPCR duplex reactions using gDNA of SK-N-AS cells as a template. Channel 1 fluorescence (FAM) is plotted against channel 2 fluorescence (HEX) for each droplet in the ddPCR duplex reactions for *MYCN/NAGK* (**A**) or *ALK/NAGK* (**B**). Red lines indicate thresholds for positivity in both channels.

Supplementary Table 1: Primers and probes used in ddPCR

Primer/Probe	Sequence (5′–3′)
MYCN forward	GTGCTCTCCAATTCTCGCCT
MYCN reverse	GATGGCCTAGAGGAGGGCT
MYCN probe	FAM-CACTAAAGTTCCTTCCACCCTCTCCT-BHQ1
NAGK forward	TGGGCAGACACATCGTAGCA
NAGK reverse	CACCTTCACTCCCACCTCAAC
NAGK probe	HEX-TGTTGCCCGAGATTGACCCGGT-BHQ1
ALK forward	AGATGGACTTGCTGGATGGG
ALK reverse	GCAGCCTCTCCCTTACCTC
ALK probe	FAM-GGCAGAGCGTTCTAAGGAGA-BHQ1
